# Enhanced Si Passivation and PERC Solar Cell Efficiency by Atomic Layer Deposited Aluminum Oxide with Two-step Post Annealing

**DOI:** 10.1186/s11671-019-2969-z

**Published:** 2019-04-18

**Authors:** Chia-Hsun Hsu, Yun-Shao Cho, Wan-Yu Wu, Shui-Yang Lien, Xiao-Ying Zhang, Wen-Zhang Zhu, Sam Zhang, Song-Yan Chen

**Affiliations:** 1 School of Opto-electronic and Communication Engineering, Xiamen University of Technology, Xiamen, China; 2 Department of Materials Science and Engineering, Da-Yeh University, Changhua, Taiwan; 3 Faculty of Materials and Energy, Southwest University, Chongqing, China; 4 Department of Physics, OSED, Xiamen University, Xiamen, 361005 China

**Keywords:** Passivated emitter and rear cell, Aluminum oxide, Atomic layer deposition, Passivation, Two-step annealing

## Abstract

In this study, aluminum oxide (Al_2_O_3_) films were prepared by a spatial atomic layer deposition using deionized water and trimethylaluminum, followed by oxygen (O_2_), forming gas (FG), or two-step annealing. Minority carrier lifetime of the samples was measured by Sinton WCT-120. Field-effect passivation and chemical passivation were evaluated by fixed oxide charge (*Q*_f_) and interface defect density (*D*_it_), respectively, using capacitance-voltage measurement. The results show that O_2_ annealing gives a high *Q*_f_ of − 3.9 × 10^12^ cm^−2^, whereas FG annealing leads to excellent Si interface hydrogenation with a low *D*_it_ of 3.7 × 10^11^ eV^−1^ cm^−2^. Based on the consideration of the best field-effect passivation brought by oxygen annealing and the best chemical passivation brought by forming gas, the two-step annealing process was optimized. It is verified that the Al_2_O_3_ film annealed sequentially in oxygen and then in forming gas exhibits a high *Q*_f_ (2.4 × 10^12^ cm^−2^) and a low *D*_it_ (3.1 × 10^11^ eV^−1^ cm^−2^), yielding the best minority carrier lifetime of 1097 μs. The SiN_x_/Al_2_O_3_ passivation stack with two-step annealing has a lifetime of 2072 μs, close to the intrinsic lifetime limit. Finally, the passivated emitter and rear cell conversion efficiency was improved from 21.61% by using an industry annealing process to 21.97% by using the two-step annealing process.

## Introduction

Passivated emitter and rear cells (PERCs) have emerged as a promising technology for both high efficiency and competitive cost in recent years. The most difference between the PERC and the traditional full-aluminum back surface field silicon solar cell is rear passivation of wafers. Considerable efforts have been made in order to improve wafer surface passivation. Minority carrier lifetimes of 0.8–8 ms have been reported for p-type floating zone wafers passivated by vacuum [1–4] or spatial atomic layer deposition (ALD) aluminum oxide (Al_2_O_3_) [5–7]. The passivation quality for p-type Czochralski wafers is lower, in the range of 0.1–2 ms [8, 9]. Spatial ALD Al_2_O_3_ have been extensively studied and applied to the industry in recent years due to their higher deposition rate (0.03–1.2 nm/s) compared to that of a conventional vacuum-type ALD (< 0.03 nm/s) [10, 11]. Trimethylaluminum (TMA) and H_2_O are the most widely used precursors as they are inexpensively volatile liquid and easy to handle. Some research groups use other precursors such as AlCl_3_ or O_3_ [12–14]. Al_2_O_3_ is currently considered to be the best passivation material due to its field effect and chemical passivation [15]. It is found that the H_2_O-based ALD process mostly leads to a silicon oxide (SiO_x_) layer at the Al_2_O_3_/Si interface, and this interfacial layer can appear after deposition or annealing [16]. Post annealing for Al_2_O_3_ films in either nitrogen or forming gas (FG) has been shown to significantly increase the wafer lifetime [12, 17]. Hydrogen in FG or Al_2_O_3_ cause hydrogenation of Si interface during annealing. The annealing temperature is typically below 500 °C, beyond which dehydrogenation occurs. However, other annealing processes for further improving passivation quality are rarely reported.

In this study, Al_2_O_3_ films are prepared on Si by spatial ALD with TMA and H_2_O as precursors. Effects of oxygen (O_2_) and FG post annealing on passivation of Si wafers are investigated and analyzed. A two-step annealing as a combination of O_2_ and FG annealing is proposed and demonstrates a higher wafer lifetime compared to the individual gas annealing process. Finally, photovoltaic performance of PERCs fabricated with industry standard, O_2_, FG, and two-step annealing are presented.

## Methods

**Fig. 1:**
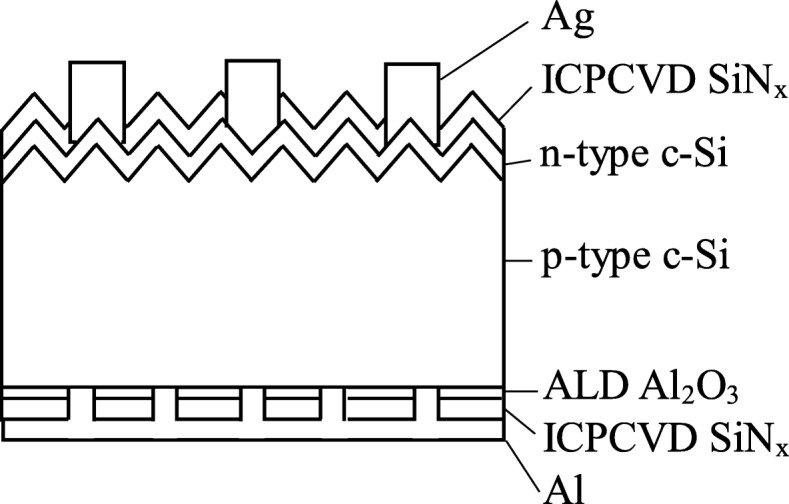
Schematic of PERC solar cells with SiN_x_/ALD Al_2_O_3_ rear passivation

P-type (100) Czochralski silicon wafers with resistivity of 1 Ω-cm and thickness of 200 μm were used as substrates. The wafers were cleaned using standard RCA process, followed by a 30-s HF dip to remove native oxide on the wafers. The Al_2_O_3_ thin films with a thickness of 18 nm were deposited using a spatial ALD system, with H_2_O and TMA as oxidant and aluminum source, respectively. The gap between gas injection heads and the movable substrate holder was about 1 mm. The detailed deposition parameters are summarized in Table 1. The temperature of the pipes was 70 °C to prevent condensation of precursors. Some of the wafers were passivated with silicon nitride (SiN_x_, 120 nm)/Al_2_O_3_ (18 nm) stack, where the SiN_x_ layer was deposited using a 13.56-MHz inductively coupled plasma vapor deposition at 120 °C with a gas mixture of ammonia (NH_3_) and tetramethylsilane (TMS). Other parameters for SiN_x_ deposition are listed in Table 2. The oxygen, FG, or two-step annealing process was performed on the samples, and the annealing parameters are listed in Table 3. The lifetime of the samples was measured by Sinton WCT-120. The capacitance-voltage (*C*-*V*) measurement was carried out on metal-oxide-semiconductor (MOS) samples by a capacitor meter (HP 4284a) at 1 MHz at room temperature. For MOS fabrication, the wafers were deposited with an 18-nm-thick Al_2_O_3_ layer and annealed. Aluminum films with a thickness of 500 nm were evaporated on both sides of the samples as electrodes. The area of the MOS samples was 1 mm^2^. The cross-sectional images of the samples were obtained using a transmission electron microscope (TEM). For PERC fabrication, a schematic of the devices is shown in Fig. 1, where the ALD passivation is only on the rear side. The wafers were textured using alkaline solution to generate random pyramids. Emitter was formed by POCl_3_ diffusion in a standard tube thermal furnace with a sheet resistance of 100 ohms/square. A SiN_x_ of 85 nm thickness was deposited on the front side of the wafer as an antireflective layer by inductively coupled plasma vapor deposition (ICPCVD). The back side of the wafer was polished by KOH solution for 3 min at 70 °C. The Al_2_O_3_ films of 18 nm in thickness were deposited using spatial ALD. An ICPCVD SiN_x_ of 120 nm in thickness was deposited on Al_2_O_3_. The samples were annealed with different annealing processes. The rear local openings with a diameter of 40 μm and a pitch of 260 μm were created by 532-nm laser scribing. Finally, a silver grid was screen printed on the front and aluminum on the rear dielectric, followed by co-firing at a peak temperature of 850 °C. The current density-voltage (*J*-*V*) curves were measured by a dual light source-type solar simulator (Wacom Co., Japan) using both xenon lamp and halogen lamp with a calibrated class A AM 1.5G simulated light spectrum.

**Table 1:** Deposition parameters of the ALD Al_2_O_3_ layer

Parameter	Value
Substrate temperature (°C)	150
TMA bubbler temperature (°C)	17.5
H_2_O bubbler temperature (°C)	30
Pressure (Torr)	760
TMA flow rate (sccm)	200
H_2_O flow rate (sccm)	500
Thickness (nm)	15

**Table 2:** Deposition parameters of the SiN_x_ layer

Parameter	Value
Substrate temperature (°C)	120
Pressure (Torr)	0.005
TMS flow rate (sccm)	15
NH_3_ flow rate (sccm)	45
Power (W)	1200
Thickness (nm)	120

**Table 3:** Parameters of O_2_, FG, and two-step annealing processes

	O_2_-600	FG-450	Two-step	
			Step 1	Step 2
Annealing gas	O_2_	5% H_2_, 95% N_2_	O_2_	FG
Temperature (°C)	600	450	600	450
Time (min)	20	20	10	10

## Results and Discussion

**Fig. 2:**
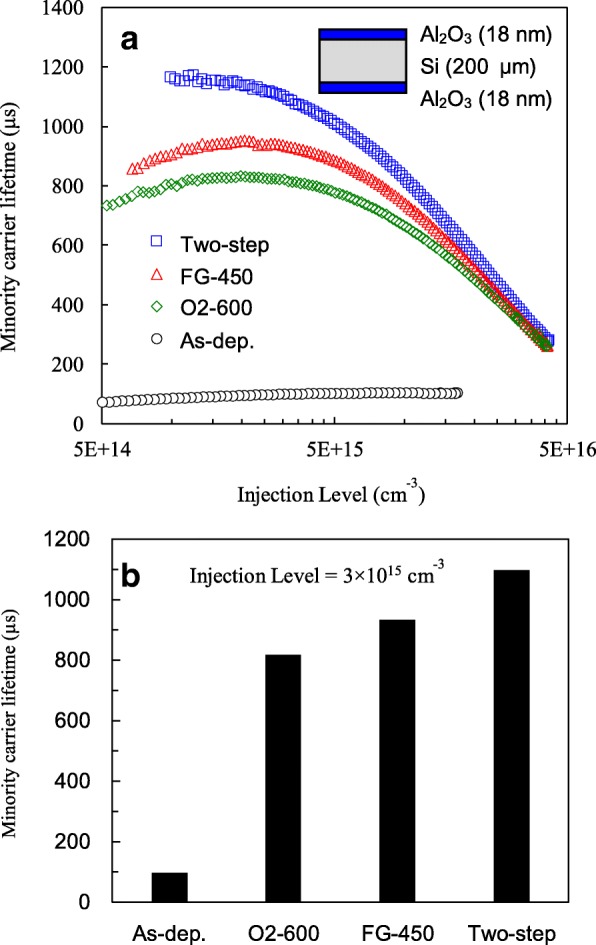
**a** Injection-level-dependent minority carrier lifetime. **b** Lifetime at an injection level of 3 × 10^15^ cm^−3^ for Al_2_O_3_/Si/Al_2_O_3_ samples with O_2_, FG, and two-step annealing

Figure 2a shows the injection-level-dependent minority carrier lifetimes of the Al_2_O_3_/Si/Al_2_O_3_ samples without and with different annealing processes. Before annealing, the minority carrier lifetime is low as below 100 μs over the whole injection level range. The lifetime greatly improves after the annealing process as a consequence of chemical passivation and field effect passivation brought by annealed Al_2_O_3_. However, the lifetime values are different in these three annealing conditions, in which oxygen annealing has the lowest curve, FG annealing has the intermediate, and the two-step annealing has the highest. The lifetime values at the injection level of 3 × 10^15^ cm^−3^ are extracted as shown in Fig. 2b. The O_2_-, FG-, and two-step-annealed samples have lifetimes of 818, 934, and 1098 μs, respectively. Note that the two-step annealing can obtain the highest lifetime only with the annealing sequence of the first step in O_2_ and the second step in FG. The reverse sequence results in a lifetime similar to that of the sample with O_2_ annealing alone. This might be because if FG annealing was performed first, the following O_2_ annealing might cause dehydrogenation. Niwano et al. reported that for a wafer terminated by Si–H or Si–H_2_ bonds, exposure to oxygen results in the replacement of the hydrogen bonds with the Si–O bonds [18].

**Fig. 3:**
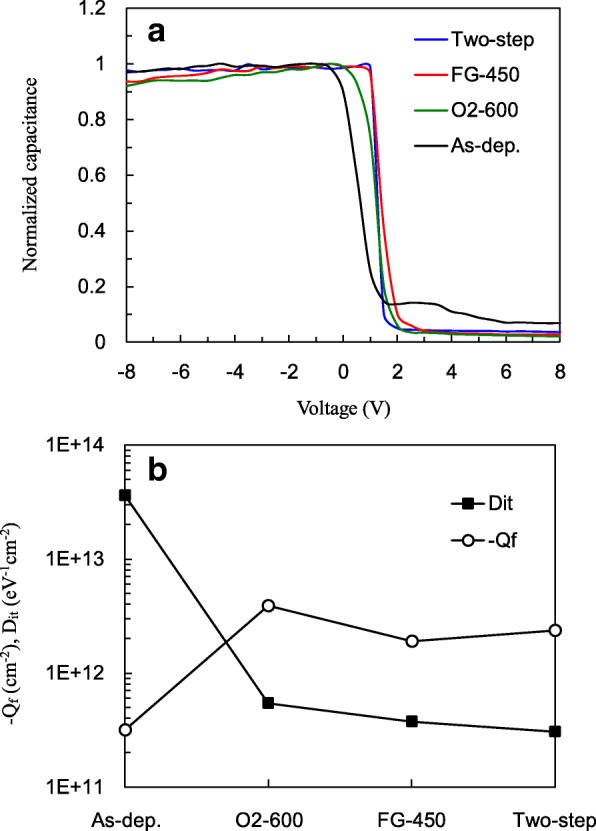
**a** Normalized *C*-*V* curves. **b** *D*_it_ and *Q*_f_ for samples with O_2_, FG, and two-step annealing

As overall passivation is governed by field effect and chemical passivation, the *C*-*V* measurement is helpful to clarify which passivation dominates in the cases of O_2_, FG, and two-step annealing. Figure 3a shows the normalized *C*-*V* curves for the samples without and with different annealing processes. The slope magnitude of the curves in the depletion region can be used as an indicator of interface defect density (*D*_it_), since the existence of interface traps causes *C*-*V* curve stretch-out [19]. The two-step annealing gives the largest slope among the others, and thus the lowest *D*_it_ is expected. To gain further information, the values of fixed oxide charge density (*Q*_f_) and *D*_it_ are extracted from the *C*-*V* curves as plotted in Fig. 3b. The *Q*_f_ is helpful for evaluating the field effect passivation and is calculated by [20]

1 $$ {Q}_f=\frac{C_{\mathrm{ox}}\left({W}_{\mathrm{ms}}-{V}_{\mathrm{fb}}\right)}{q\ A} $$

where *C*_ox_ is the accumulation oxide capacitance, *W*_ms_ is the work function difference between semiconductor and electrode (in this case − 0.9 V), *V*_fb_ is the flat band voltage, *q* is the electron charge, and *A* is the area of the MOS devices. The *Q*_f_ is − 3.2 × 10^−11^ cm^−2^ for the as-deposited sample. *Q*_f_ at this level leads to weak field effect passivation [21]. All the annealed samples elevate *Q*_f_ to the level of 10^12^ cm^−2^. It is seen that the O_2_ annealing gives the highest *Q*_f_ of 3.9 × 10^12^ cm^−2^, the two-step annealing gives the intermediate *Q*_f_, and the FG annealing gives the lowest *Q*_f_. On the other hand, *D*_it_ value estimated by the Terman method [22] is also shown to evaluate chemical passivation. The as-deposited sample has a *D*_it_ of more than 10^13^ eV^−1^ cm^−2^. It reduces to 5.4 × 10^11^ eV^−1^ cm^−2^ for O_2_ annealing, 3.7 × 10^11^ eV^−1^ cm^−2^ for FG annealing, and 3.1 × 10^11^ eV^−1^ cm^−2^ for two-step annealing. Thus, by comparing O_2_ and FG annealing, it is found that O_2_ annealing has the better field effect passivation, whereas FG has the better chemical passivation. The former might be linked to the interfacial SiO_x_ growth. Unlike FG annealing which is performed at a relatively low temperature and with lack of oxygen, O_2_ annealing is expected to have an improved SiO_x_ interfacial layer growth. This could increase the possibility of Al substitution for Si at the Al_2_O_3_/SiO_2_ interface, which is regarded to be one possible origin of negative fixed charges [23]. Considering the two-step annealing, the intermediate *Q*_f_ is reasonable as a combination of O_2_ and FG annealing. However, its *D*_it_ value is lower than that of the FG annealing. This is explained by the additional contribution by the higher quality of the interfacial oxide layer due to the first-step O_2_ annealing. Some studies also reported that a denser SiO_x_ results in a better passivation [24]. The lower *D*_it_ in two-step annealing sample can also be attributed to the hydrogenation improvement of silicon surface induced by the hydrogen in Al_2_O_3_ film.

**Fig. 4:**
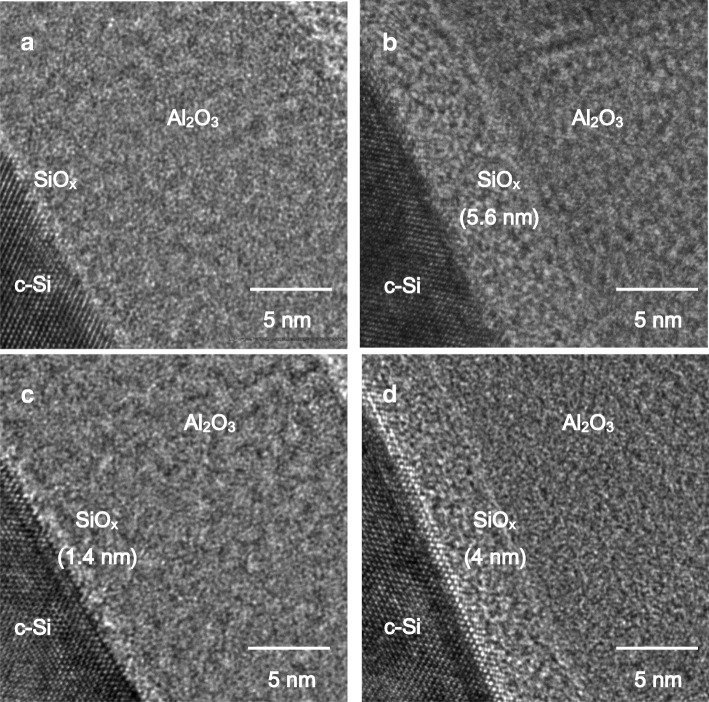
Cross-sectional TEM images for samples **a** without annealing and with **b** O_2_, **c** FG, and **d** two-step annealing

Figure 4 shows the cross-sectional TEM images of the samples without and with different annealing processes. Before annealing, a SiO_x_ interfacial layer between Si and Al_2_O_3_ is observed although the interface is not clear. This might be because H_2_O was used in the first-half ALD cycle. For O_2_ annealing, the interfacial layer thickness increases to 5.6 nm, due to annealing at a high temperature (600 °C) and in oxygen ambient. It has been reported that oxygen has a very small diffusion coefficient in Al_2_O_3_ (~ 10^−38^ cm^−1^ at 600 °C) [25], and thus, it is unlikely for oxygen to diffuse through the Al_2_O_3_ layer to reach the Si interface. Instead, ambient oxygen interchanges with the oxygen in Al_2_O_3_, creating a mobile oxygen that can repeat the interchange process in the deeper Al_2_O_3_ region until the oxygen reaches the Si interface [26]. The sample annealed in FG shows a clearer interface with a very thin SiO_x_ interfacial layer of 1.4 nm, which is similar to other research groups performing the annealing process in N_2_ or FG [16]. This evidences that FG annealing limits the interfacial layer growth. The two-step annealing shows an intermediate SiO_x_ interfacial layer thickness of about 4 nm, as a consequence of the reduced time of the O_2_ annealing.

**Fig. 5:**
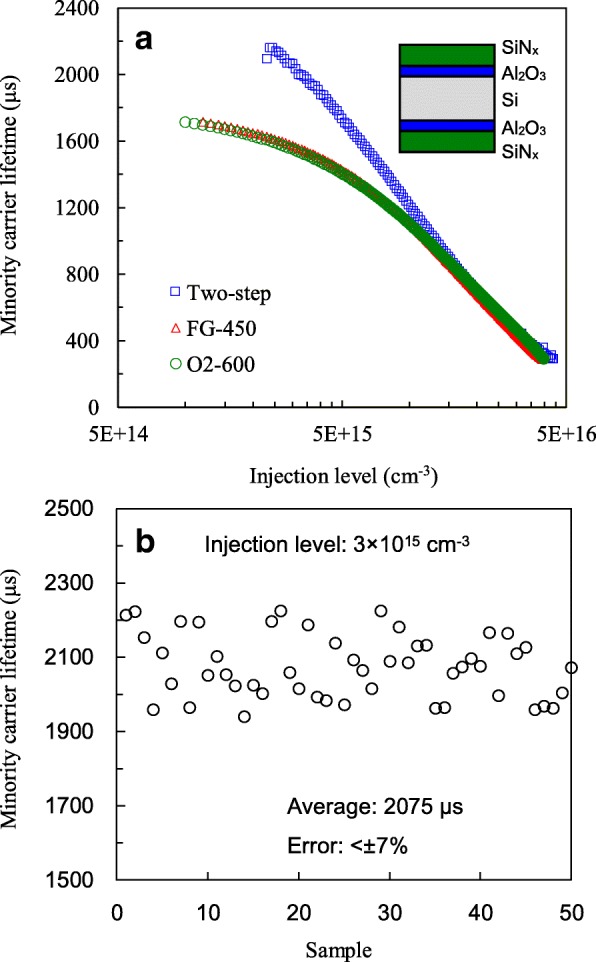
**a** Injection-level-dependent minority carrier lifetime of SiN_x_/Al_2_O_3_-passivated samples with O_2_, FG, and two-step annealing. **b** Lifetime at an injection level of 3 × 10^15^ cm^−3^ for 50 samples with two-step annealing

Figure 5a shows the injection level-dependent minority carrier lifetime of the SiN_x_/Al_2_O_3_-passivated wafers without and with different annealing processes. The lifetimes at the injection level of 3 × 10^15^ cm^−3^ are 1569, 1579, and 2072 μs for O_2_, FG, and two-step annealing, respectively. The improvements are related to that the plasma chemical vapor-deposited SiN_x_ films may contain certain amounts of hydrogen depending on the deposition process parameters. During the annealing process, some of the hydrogen would move towards the Si interface, and this enhances the Si interface hydrogenation [27]. As reported in literature [6, 28–30], the lifetime of SiN_x_/Al_2_O_3_-passivated p-type CZ wafers is in the range of 0.1–2 ms. The optimal temperature of post-deposition annealing either in nitrogen or in FG is around 400–500 °C. In this work, the SiN_x_/Al_2_O_3_-passivated CZ wafer annealed in FG shows a lifetime of 1579 μs and an optimal annealing temperature of 450 °C, which are in accordance with the reported values. However, this optimal temperature is limited by the hydrogenation of the silicon interface. From the viewpoint of the silicon oxide interfacial layer, this layer might have different optimal temperature as high temperatures generally improve qualities of silicon oxide films. Thus, the two-step annealing could optimize both of the interfacial oxide quality and silicon interface hydrogenation, and leads to a higher lifetime of 2072 μs compared to the case of forming gas single-step annealing. To investigate the reproducibility, 50 samples with two-step annealing were prepared, and their minority carrier lifetime is shown in Fig. 5b. The samples have lifetime values ranging between 1939 and 2224 μs. The average value is 2075 μs, and the error is within ± 7%. The intrinsic lifetime limit of the wafer used in this study is about 2300 μs, calculated by using the Richter parameterization [31]. Thus, the two-step annealing yields a lifetime close to the lifetime limitation and demonstrates excellent interface passivation. For other ALD, a silicon oxide interfacial layer between Al_2_O_3_/Si is also found, and the two-step annealing should be able to improve the passivation quality of Si wafers. AlO_x_/SiN_x_ is necessary as the silicon nitride not only enhances passivation but also increases rear reflectance and protects AlO_x_ from a high-temperature cofiring process for PERC fabrication.

**Fig. 6:**
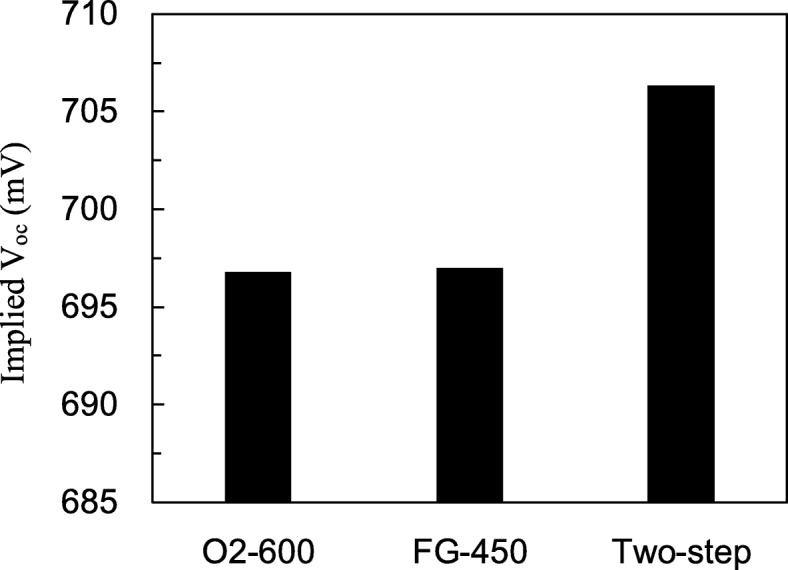
Implied *V*_oc_ of the SiN_x_/Al_2_O_3_-passivated samples with O_2_, FG, and two-step annealing

Figure 6 shows the implied open-circuit voltage (*V*_oc)_ for the SiN_x_/Al_2_O_3_-passivated samples with different annealing processes. For p-type wafers and long diffusion lengths, the implied *V*_oc_ can be written as

2 $$ \mathrm{implied}\ {V}_{\mathrm{oc}}=\frac{kT}{q}\ln \left(\frac{\Delta  n\ \left({N}_A+\Delta  n\right)}{{n_i}^2}\right) $$

where *k* is the Boltzmann constant, *T* is the absolute temperature, *n*_*i*_ is the intrinsic carrier concentration, *N*_*A*_ is the acceptor concentration, and ∆*n* is the excess carrier concentration measured at one-sun light intensity by the WCT-120 Sinton lifetime tester. It can be seen that the O_2_- and FG-annealed samples have similar implied *V*_oc_ values, which are 696 and 697 mV, respectively. The two-step annealing has an implied *V*_oc_ of 706 mV.

**Fig. 7:**
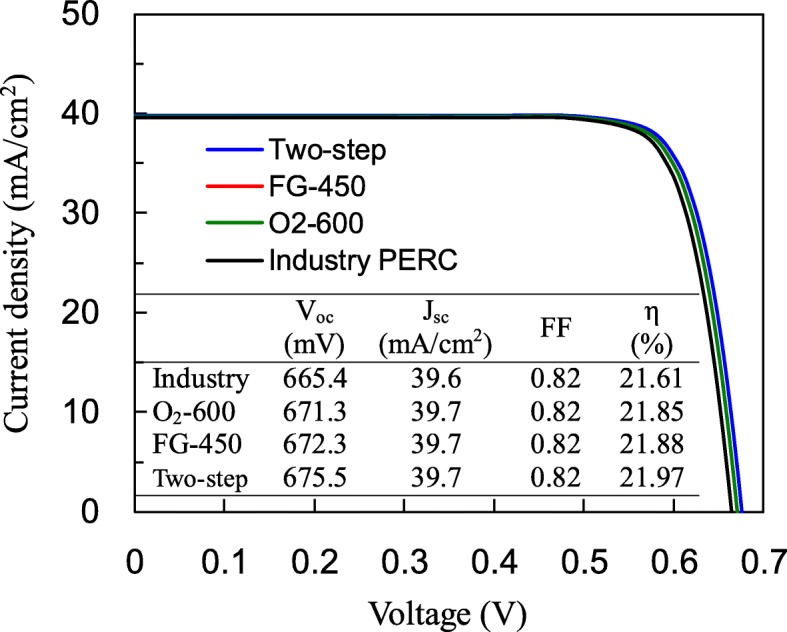
Current density-voltage curves and photovoltaic performance of PERCs with industry standard fabrication, O_2_ annealing, FG annealing, and two-step annealing

**Fig. 8:**
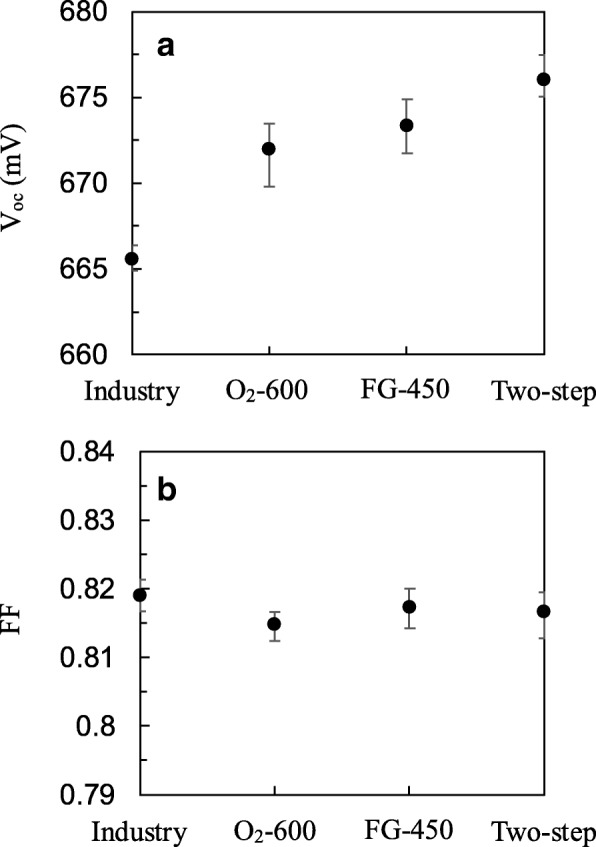
Mean value and distribution range of **a** *V*_oc_ and **b** FF for PERCs with different annealing processes

Figure 7 shows the *J*-*V* characteristics and photovoltaic parameters such as *V*_oc_, short-circuit current density (*J*_sc_), fill factor (FF), and conversion efficiency (*η*) of the fabricated PERCs with different annealing processes. The performance of an industrial PERC is also shown for the purpose of comparison. The industry PERC was fabricated under identical conditions but no additional annealing process was used, since the Al_2_O_3_ layer was annealed during the SiN_x_ deposition at 400 °C. Note that in this study, during the annealing processes, the front side was placed downward and made contact to a wafer holder. The front SiN_x_ layer was not exposed to the annealing gases, and thus, the influence of the front SiN_x_ layer might be insignificant. The industry PERC shows the lowest *V*_oc_ of 665.4 mV among the others. This could be attributed to its lower wafer lifetime of 797 μs at the injection level of 3 × 10^15^ cm^−3^. The *V*_oc_ value improves to 671.3 mV for O_2_ annealing and 672.3 mV for FG annealing. The two-step annealing further increases *V*_oc_ to 675.5 mV, which is an improvement by about 0.6% compared to one-step annealing, or by 1.5% compared to the industry one. There is no much difference in *J*_sc_ and FF between the PERCs. The two-step annealing exhibits the best conversion efficiencies of 21.97%, which is 0.36%abs higher than industry PERC. Finally, five PERCs were fabricated for each annealing process. The mean value and distribution range of *V*_oc_ and FF are shown in Fig. 8a and b, respectively. The PERCs with the two-step annealing show *V*_oc_ of 675–677.5 mV with a mean value of 676 mV, and FF of 0.813–0.819 with a mean value of 0.816.

## Conclusion

The Al_2_O_3_ films are prepared using atomic layer deposition, followed by O_2_, FG, or two-step annealing. Comparing O_2_ annealing with FG annealing, the former yields a thicker SiO_x_ interfacial layer and the higher *Q*_f_ density of − 3.9 × 10^12^ cm^−2^, indicating a superior field effect passivation. The FG annealing shows the lower *D*_it_ of 3.7 × 10^11^ eV^−1^ cm^−2^ resulting from the hydrogenation of the Si interface. The two-step annealing combines the advantages of these two annealing processes and has an intermediate *Q*_f_ and the lowest *D*_it_ of 3.1 × 10^11^ eV^−1^ cm^2^. The SiN_x_/Al_2_O_3_-passivated samples with the two-step annealing demonstrate a minority carrier lifetime of 2072 μs, close to the intrinsic lifetime limit. For the PERC fabricated with the two-step annealing, *V*_oc_ of 675.5 mV and conversion efficiency of 21.97% can be obtained, which respectively have increases of 10 mV and 0.36%abs as compared to those of the industry PERC.

## Abbreviations

Al_2_O_3_: Aluminum oxide
ALD: Atomic layer deposition
*C*-*V*: Capacitance-voltage
*D*_it_: Interface defect density
FF: Fill factor
FG: Forming gas
*J*_sc_: Short-circuit current density
*J*-*V*: Current density-voltage
MOS: Metal-oxide-semiconductor
NH_3_: Ammonia
O_2_: Oxygen
PERC: Passivated emitter and rear cell
*Q*_f_: Fixed oxide charge
SiN_x_: Silicon nitride
SiO_x_: Silicon oxide
TEM: Transmission electron microscope
TMA: Trimethylaluminum
TMS: Tetramethylsilane
*V*_oc_: Open-circuit voltage
*η*: Conversion efficiency
